# “An illustrative socio-biological cascade of the concentric model for health-bound networks: social determinants, social support and biological responses”

**DOI:** 10.3389/fsysb.2026.1920622

**Published:** 2026-09-07

**Authors:** Michele Battle-Fisher

**Affiliations:** Equitas Health Columbus, Columbus, OH, United States

**Keywords:** complexity, health, network science, social support, systems biology, systems science, systems thinking

## Abstract

**Introduction:**

Health has been associated with social support. Supported patients with complex health issues have a better chance of living healthier, longer lives and enjoying a better quality of life. Research indicates that physical, mental, and emotional health are connected to both biological and social determinants.

**Methods:**

This paper proposes a conceptual, society-to-biology model, the Concentric Model for Health-Bound Networks, that explores the complexity of social support and integrates the foundational principles of systems biology and systems thinking. This transdisciplinary model conceptualizes the sources of support for patients requiring care as dynamic, concentric social networks. The model is informed by current understanding of the interdependence between disease and social support. The Concentric Model for Health-Bound Networks attempts to clarify the configuration of care-intensive social support by maintaining and transforming diverse, overlapping webs of support that change dynamically. Drawing upon network science and systems thinking, this model examines how these approaches apply to the capacity of social support to improve health outcomes and inform clinical practice.

**Results:**

While the Concentric Model for Health-Bound Networks is untested, complex, care-intensive social support requires dynamic models that adapt to complexity. Via a transdisciplinary socio-biological approach called the Socio-Biological Cascade, feedback may co-occur across the Social Determinants of Health, social support, and biological processes. Type 2 diabetes management, with its overlap of complex biological processes and individualized health-related requirements, serves as a theoretical example of how social support informs the Socio-Biological Cascade and its possible implications for health.

**Discussion:**

Changes in the composition, quality, change over time, and structural integrity of health-bound support networks may mediate or modify relationships between social determinants of health and biological stress, inflammatory, and metabolic responses, thereby affecting chronic disease outcomes. It is hypothesized that the dynamics of patient-centered social support, informed by the Social Determinants of Health and applying the Socio-Biological Cascade and Concentric Model for Health-Bound Networks, can elucidate complex health outcomes.

## 1 Introduction

The presence of disease is related to Social Determinants of Health (SDOH) that affect health status (Costa et al., 2026; McDade and Harris, 2022). Macro-level SDOH may affect a patient’s biological states and the presence of disease. As said by McDade and Harris, “Explanations for disease are located “inside the body,” with little, if any attention given to a causal role for social or other contextual factors” (McDade and Harris, 2022: 4). According to Krieger, biology is ecosocial, meaning the interaction of social factors and determinants is tied to disease (Krieger, 2020). Systems biology is not divorced from the wickedness of human health. Krieger describes embodiment-bodies tell “stories” of complex social-biological connections (Krieger, 2005). The relationship with disease becomes more than a biological one; it is a manifestation of the biosocial interactions of social factors and physiology embedded in and expressed through our bodies. The social and biological mechanisms are concentrically related, forming a socio-biological relationship. An example of the social and biological connection is the possible role of mediating social support for patients with complex needs.

Lifetime environmental exposures, which can lead to physiologically distinct experiences and manifestations of chronic diseases, are collectively referred to as the exposome (Wild, 2005). While Wild concedes that the exposome may not be fully quantifiable, the implications of the interaction between physiological and social exposures specific to the patient over time are fundamental to understanding chronic disease in a personalized, systemic manner (Wild, 2005). Zia and colleagues discuss the debate over genetic predisposition, further accounting for environmental determinants of illness alongside genetics (Zia et al., 2026). According to Lovejoy, for human biology, “The environment (of the exposome) includes the systems in which we live and work, such as healthcare, government, and culture (including views of race, gender, social class, and physical ability)” (Lovejoy, 2024: 2). Lovejoy noted the need for greater precision in measuring the nature of interdependence in exposome factors within the social environment (Lovejoy, 2024). A systemic method and approach are needed to uncover and describe exposome factors in an innovative way. In the exposome, disease is personalized, with a distinct trajectory shaped by physiological and social factors. The unique feature of the external exposome is the human connection to biology in a complex ecosystem. For systems biology to be connected to SDOH, a mediator is required. One such mediator could be social support.

According to Winkelmann and colleagues, the phenomenon of a social cascade, which may resemble an ecological trophic cascade, exhibits social tipping points driven by human agency (Winkelmann et al., 2022). The trickle-down effect in this analogy is helpful in socio-biological contexts, as change in the ecosystem may alter the system’s structure. A Socio-Biological Cascade can be viewed as a set of interconnected triggered processes, with feedback loops between biological and social factors that affect health. While not implying or establishing causation, it is hypothesized that SDOH are connected to mediators, such as social support, that link to biological processes. It is proposed that socio-biological cascades may co-occur, with elements of this Socio-Biological Cascade connected through feedback mechanisms. For entities to be considered organizationally a cascade, elements in an ecosystem must be activated and connected in ways that trigger reactions across the ecosystem. When human irrationality in choices and reactions within an ecosystem is combined with biological mechanisms, the sequential, top-down interactions within an ecosystem become less predictable and may be amplified. Socio-Biological Cascade reactions may be hard to predict, though it can be anticipated that changes at one level may influence or propagate to the other levels.

The Socio-Biological Cascade describes a complex biological and social progression that may shape individual health outcomes. This framework begins with influential external environmental factors, known as SDOH, such as economic stability and structural disadvantages, which can shape a person’s experience with disease. These initial conditions may directly influence the quality and structure of support networks, including family, peers, and community resources. In turn, the strength of these social connections may influence how a person’s internal biological systems manage chronic stress. When social support is lacking, the body may undergo neuroendocrine and immune shifts that trigger inflammation and metabolic dysfunction.

**FIGURE 1:**
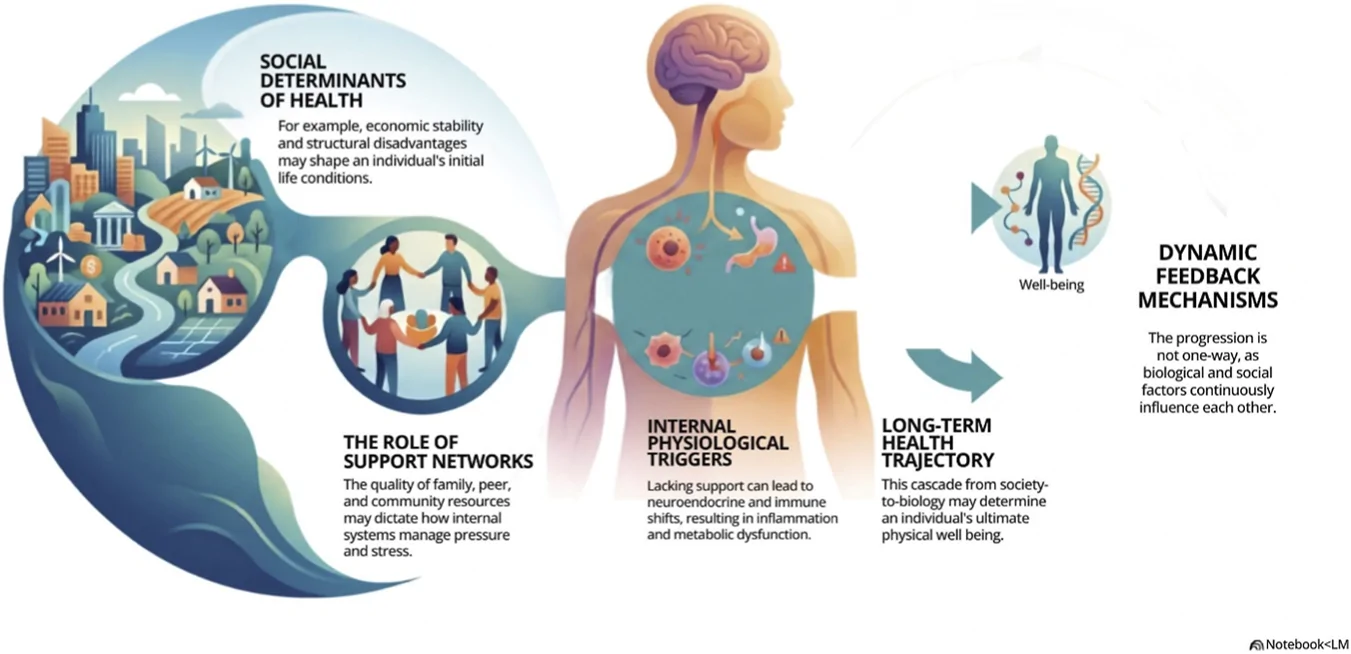
The Socio-Biological Cascade. Figure created using AI (Gemini Notebook).

Figure 1 illustrates the proposed complex progressive relationships of systems biology, social support, and select SDOH. The selection of SDOH in the figure is representative and not fully exhaustive. Social Determinants of Health, social support systems, and systems biology may be interconnected and dynamically influence one another. These elements could work together to influence several integrated health outcomes: lowered risk of chronic disease, enhanced mental wellbeing, increased resilience, and the promotion of health equity. It is hypothesized that other mediating factors could be substituted for social support within the Socio-Biological Cascade. The proposed Socio-Biological Cascade in Figure 1 applies social support networks, possible SDOH, systems biology, and feedback mechanisms.

There may be a synergistic relationship between social support and SDOH in individualized experiences with disease. For instance, individuals with economic means may have better health outcomes. For example, social connection and avoidance of social isolation for those with the most extreme exposure to stressors may act as a protective buffer against some consequences that may lead to economic instability (Moskowitz et al., 2013). Moskowitz and colleagues note differential effects of social support, which work on a gradient for under-resourced populations (Moskowitz et al., 2013). According to Moskowitz et al.‘s study, “Social support (did) not buffer the effects of stressors on health uniformly for individuals living in conditions of urban poverty” (Moskowitz et al., 2013: 175). This finding highlights the need for careful nuance in accounting for population variations when analyzing social support, particularly in under-resourced populations.

The connection between social connection and physical health is more likely bidirectional and cyclical in some cases (Holt-Lunstad, 2024). By translating findings of social connection to physical health, this paper argues that the existence of social networks for support can be leveraged to begin to investigate potential mechanisms of social connections to health (Holt-Lunstad, 2024). Framing social connections more specifically as a source of bidirectional and cyclical health support may help to account for both health-related and more general sources of social support.

For example, SDOH and their accompanying stress are said to be associated with allostatic load, in which stress wears and tears on the body (Guidi et al., 2021; Hayward et al., 2000). One hypothesis of note is the “weathering” hypothesis, which is tied to the structural disadvantage of poverty, in which wear and tear is shown as a negative effect on allostatic load of racialized bodies due to racism and concentration of disadvantage, factors which are not explained by poverty, and is experienced across income levels (Geronimus et al., 2006).

Leschak and Eisenberger posit a connection between social connection via social ties and evolutionary biological response, asserting that evolutionary mechanisms support “what kinds of immune processes might be needed … and to redirect energy and resources to address those critical needs” (Leschak and Eisenberger, 2019). The implications for the Socio-Biological Cascade, as presented, are:

1. Social support is connected to varying degrees to physiological changes that affect health outcomes.
2. SDOH are connected and affect social support, which then may affect physiology and disease.

There may be physiological and social implications of this Socio-Biological Cascade for detecting and mitigating stress within social networks. The hypothalamic-pituitary-adrenal (HPA) axis is a principal biological pathway involved in stress responses and the secretion of the adrenal stress hormone, cortisol, which is activated during fight-or-flight and plays a key role in regulating chronic stress. According to Dunlavey, the HPA is connected to evolutionary survival, longevity, biological development, and homeostatic maintenance through a combination of physiological systems (Dunlavey, 2018). While stress is a normal, expected physiological state when acute, it becomes problematic for the body when it is chronic and uncontrolled, potentially leading to a plethora of chronic diseases.

In the Socio-Biological Cascade, biological mechanisms may be effectively constrained or amplified by social support systems. In other words, having other people as support may affect a patient’s physiology and disease state. A possible social response to chronic stress is to intervene in the Socio-Biological Cascade through social support systems, which may increase the likelihood of identifying a social leverage point within them to improve health outcomes.

Some evidence has shown a bidirectional relationship between social relationships and immune response (Leschak and Eisenberger, 2019). According to a review by Leschak and Eisenberger, there is social variation in inflammation and antiviral immunity, forming two possible pathways through which socio-biological interaction occurs (Leschak and Eisenberger, 2019). Leschak and Eisenberger presented a novel evolutionary framework, one that accounts for the biological effects of social isolation and embeddedness in social networks (Leschak and Eisenberger, 2019). The review findings were:

1. “Adverse social experiences” may bring about inflammation while suppressing immune function. The converse may also be true, as the presence of social connection and increased pathogen exposure may lead to improved immune responses.
2. The constant presence of inflammatory responses may be linked to adverse physiological reactions, possibly leading to worse health outcomes. (Leschak and Eisenberger, 2019: 2).

A longitudinal study of United States. adults investigated the relationship of the nature of social support to the risk of inflammation, illustrated by five physiological markers tied to immune response (C-reactive protein, interleukin-6, fibrinogen, E-selectin, and intracellular adhesion molecule-1) (Yang et al., 2014). The Yang et al. findings were:

1. Close kin and friends had a modest protective effect on the risk of inflammation.
2. “Social strain” as well as the presence of family and friends substantially increased risks of inflammation.
3. In general, social strain had a stronger negative association with inflammation, while social support had a lesser positive effect on the risk of inflammation. (Yang et al., 2014).

Overall, elevated inflammation burden was found to suggest that social strain from social networks with a variety of alters is a risk factor (Yang et al., 2014).

According to the buffering hypothesis, fostering and leveraging supportive relationships may serve as a protective buffer against highly stressful events, thereby influencing health outcomes and biological responses to high stress (Kroenke et al., 2013). Another approach is the main-effect model or direct effects, which posits an association between “the overall benefit of support” and wellbeing (Kroenke et al., 2013). Cohen and Wills noted that both buffering and main effect/direct effects may be associated with better wellbeing, but in different complex social situations (Kroenke et al., 2013). While the buffering hypothesis is applied during high stress to understand its biological and emotional effects, the main effects, or direct effects, have a broader scope of application, including non-stressful events. The buffering hypothesis postulates a direct relationship between stress and support. The main-effect measurement coincides with the presence of and embeddedness in large social networks (Kroenke et al., 2013). Main effects and stress buffering are linked, may occur together, and are therefore not mutually exclusive (Rodriguez et al., 2019). The social support structure and biological function are explored indirectly in the model to discern the complexity of dynamic social support’s effects on a patient’s biological reactions and social connections. The presence or absence of support may relate to contending with both chronic, heightened stress and generalizable, unremarkable stress.

Social safety is related to the composition and actions inherent to a patient’s social support within networks. For example, chronic stress can be mitigated with social support. The Social Safety Theory states that social connections are fundamental to controlling and buffering psychophysiological stress, which can lead to diminished social safety, which can lead to adverse health outcomes (Slavich, 2020). Slavich notes that the immune system, which is affected by stress, cannot directly detect the influence of social factors; it is the brain that plays that role (Slavich, 2020). But there is also a social brain. In terms of the power of the social brain linked to larger primate brains, complex social bonds require complex evolutionary neural pathways to navigate them successfully (Dunbar and Shultz, 2007). There is not only safety in numbers in a social network, but there is perceived and actual safety in the quality of those bonds. The physiology requires buffering the effects of stress. And social support is only possible with people who can support situations that ensure social safety.

There may be a Socio-Biological Cascade, a society-to-biology progression, in which a trigger, such as SDOH via social support mechanisms, may lead to biological reactions that are both socially related and disease-related. Moreover, when social and biological factors interact with the environment in interdependent ways, small changes in the social and/or biological environments may lead to widespread, notable, non-linear systemic effects. It is hypothesized that changes in the composition, quality, and temporal changes of health-bound social support mediate or modify the relationship between SDOH and biological processes, such as stress response, inflammation, and metabolic responses, thereby affecting chronic disease outcomes.

## 2 Social network analysis and deciphering complex health outcomes

Social Network Analysis (SNA), when used with the intent to uncover real-world complexity, reveals how health is negotiated, perceived, and realized. According to Blanchet and James, there is a missed opportunity to explore health systems through SNA to unpack the complex interplay between personal illness and public health (Battle-Fisher, 2015; Blanchet and James, 2012). The areas of opportunity missed, according to Blanchet and James, are:

1. Exploring the dynamic relationship between the system and the network, focusing on the embeddedness of systemic structure on health.
2. Uncovering structural factors that affect health systems.
3. Discovering the connection of multiple, interdependent concentric systems. (Blanchet and James, 2012).

The use of SNA, which illustrates and measures real-world social complexity, lends itself to a fusion of qualitative and quantitative methods, which provide a holistic view (Battle-Fisher, 2015; Yousefi Nooraie et al., 2020; 2021). Social Network Analysis (SNA) is more than a method of discovering interdependence and connections among people and institutions. The mathematics of SNA should be translated into a narrative that illustrates what the graphs and numbers represent in the real world (Yousefi Nooraie et al., 2021). Social support matters to health outcomes, and this dynamic support can be mathematically measured and qualitatively defined to inform patient care.

Social support can come from social networks, or webs of people connected to a person or ego in a network. There is a distinction between social support and social networks, but they are linked (Drageset, 2021). Social support is the primary functional aspect of social networks (Drageset, 2021). The conventional operationalization of social support often fails to account for social networks and their structural dynamics (House et al., 1988). Structural social support is defined as the measurement of the social network’s characteristics (Drageset, 2021). Functional social support is the qualitative “appraisal” of that structural support (Drageset, 2021). Social resources, fundamental to positive health outcomes, are “embedded into ties” (Lin, 2000). Every social interaction has goals, and people are vested with the choice of acting on them or not. These network-based social interactions are driven by the pursuit of “needed and useful” resources for exchange (Perry and Pescosolido, 2010).

Social support is necessary to deal with complex matters such as navigating illness and disease. Social support requires people who share a common purpose. The smallest unit in a network is the dyad, or two people linked with that purpose. Insufficient attention has been paid to understanding dyadic relationships over time from the perspective of alters (support people) within ego, patient-centered networks (Offer et al., 2026). McCarty posited that by extrapolating beyond attribute-based measurements in social networks, the structure of the network should be explored to more fully understand the state of the network (McCarty, 2002). For example, Yousefi-Nooraie et al. presented the fused method, which iteratively uses SNA, framed by thematic, qualitative methods and conventional SNA, to discern the state of caregiving support networks (Yousefi Nooraie et al., 2021). The story of social support does not end with the patient’s network characteristics. The implications of this gap in the literature are that the history of linked relationships cannot be fully described or understood without intentionally conceptualizing, measuring, and applying these findings to improve health on a nonlinear scale.

According to Finfgeld-Connett, there is a distinction between care and social support (Finfgeld-Connett, 2007). Care is provided by healthcare providers, and social support is provided by non-healthcare providers, such as kin and friends (Finfgeld-Connett, 2007). The interdependencies between health and disease should be the focus of clinical care to prevent adverse outcomes (Sturmberg and Lanham, 2014). Physical wellbeing appears to be supported by the caring mechanism, with social support emphasizing its focus on the patient’s specific needs (Finfgeld-Connett, 2007). While Finfgeld-Connett argues that caring and social support are, to an extent, processes that do not occupy the same social space, their interdependence highlights the multiplex role of diverse support within social networks, and that the spaces and resources for caring and social support overlap (Finfgeld-Connett, 2007). The resources embedded in relationship ties may differ between carers and support people, but the multiplex roles people occupy across the whole support network may also differ. Relationships are unique, and connections change dynamically, depending on the support available across social levels of involvement, patient needs, and social circumstances. For the remainder of this paper, the category of support person will include the formal carer, as well as a formal or informal support person. The commonality of carers and supporters will be captured through network positions and network characteristics that formal and informal support people of all kinds play in the patient’s health.

An integral part of care-intensive social support is quality of life (QOL), which may be linked to one’s social support networks. The type, frequency, and source of social support matter in different ways (Rezaei et al., 2019). For example, social isolation has been linked to lower self-rated health (Cornwell and Waite, 2009). Additionally, larger social networks, embedded with social support, are associated with improved QOL following cancer diagnosis (Kroenke et al., 2013). Diverse, “locally integrated” networks, consisting of kin and non-kin, are protective against adverse mental health outcomes among older adults (Wenger, 1996). Conversely, “private restricted” networks with little to no informal kin and non-kin support are most at risk for adverse mental health outcomes among older adults (Wenger, 1996). People with mental and physical health concerns tend to have smaller, less dense, and under-resourced social support network structures and composition (Luke and Harris, 2007).

## 3 Network-based connection of health to a socio-ecological context

There is an important distinction between social stress and more general, less specific stress on social networks and their ties. The effect of one’s social environment, which is distinct from social stress, is often deemed to be stronger in influence than other forms of stress (Wood and Bhatnagar, 2015). The subjective appraisal of defining and understanding stressors differs in valence from the objective, constructed understanding of stressors (Almeida, 2005). Health, defined as mental health, chronic health problems, and acute disease, is susceptible to temporal and dynamic vulnerability and individual reactions to stressors (Almeida, 2005). When applied to social networks, this vulnerability of stress and subjective appraisal of stressors may be pronounced as social ties are susceptible to social stress. The context and influence of social stress on relational dynamics underscore that networks are context-dependent, and the tie-based relationship between nodes may stretch and snap or strengthen under both external and internal societal pressures (Battle-Fisher, 2015).

Tie formation varies across different social contexts (Fuhse and Gondal, 2024). In addition to nodes that are risk factors, nodes, or people in a network, can represent people connected by spatial proximity or propinquity in social relationships. Gaining resources, such as social support and social capital within a social network, relies on maintaining the integrity of its network ties. Nodes in social networks may sever or strain ties dynamically for various reasons. The effect of societal stress on the integrity of network ties is an understudied area in SNA. This fluidity in social relationships is tied to the social context in which these relationships form, change, and dissolve. The fluid reality of connections requires a reimagining of social support.

A social elastic tie, exhibiting the physics of a rubber elastic, is a link between dyadic nodes that may be susceptible to endogenous social and organizational stressors, as well as stress from the external environment (Battle-Fisher, 2015). This exposure to the internal and external exposomes can subsequently compromise the social elastic tie, affecting its integrity. Social elastic ties may dynamically respond to inherent differences in power and social capital embedded within the resources associated with the ties. Social stressors introduce unique social outcomes, which can affect relationships and undermine the integrity of a person’s social network and its connections. It is hypothesized that the social elastic tie responds to the acquisition of power, the allocation of resources, and the possession of social capital. The social network tie reflects the difficulty of maintaining and nurturing network connections, which are socially bound and locally influenced. Network attributes, such as homophily, reciprocity, and transitivity, provide some context, and the ties offer that context in terms of power and social capital (Fuhse and Gondal, 2024). The social elastic tie builds upon the understanding of social meaning and culture in differing contexts of tie formation, which is associated with relational sociology (Fuhse and Gondal, 2024).

Context-sensitive influences and environments that limit community integration and engagement affect social elastic ties within a diverse social network with unmet health needs. A tie can represent societal power and influence in strong connections, which are better equipped to handle complexity (Valente, 2010). According to Diez Roux, “Biology interacts with environments and individuals interact with each other and with environments over time” (Diez Roux, 2011:1627). Understanding the contexts that shape environments affecting patients is crucial for identifying health inequities and gaining insights to achieve better health outcomes. It is assumed that embedded social conditions are connected to an ecological environment that sustains them. The aim of using a network-based socio-ecological perspective in the model is to identify ways to reduce exposure to environmental stressors in the exposure to the exposome and to leverage connections within social networks to improve health. The integrity of social elastic ties may be a key factor in maintaining social connectivity, securing relationships, and leveraging resources. Consequently, systems may house different networks with varying resources and opportunities for life. McFarland et al. noted that the social environment does not adapt, for instance, to changes in societal relationships (McFarland et al., 2014).

People transform the social environment through “(mobilization) to change institutional structures, practices or rules” (McFarland et al., 2014: 1090). In social networks, network ecology is an ecological framework that “(identifies) how the proximal environment shapes social networks by focusing on interactions and social relations, and how these interactions and relations in turn shape the environment in which social networks form” (Doehne et al., 2024a: 1). The ecological context influences the formation of ties and the fostering of relational dynamics (Doehne et al., 2024b). According to Doehne et al., network ecology helps contextualize the environment in which networks sustain relationships (Doehne et al., 2024b). The context and effects of societal stress on relationship dynamics underscore that connections are contingent upon specific situations in the exposome.

Offer and colleagues presented a novel network science approach, called the Alter-Trajectory Approach (Offer et al., 2026). This approach has the potential to explore the relationship between the patient (ego) and their support people (alters). According to the Alter-Trajectory Approach,

“It matters, for example, whether an alter who is no longer listed in the network had previously played a long-standing, central role in ego's network, or whether they were peripheral – or even just met – prior to being named. Put more technically, egocentric (personal) networks … exhibit higher-order dynamics in which the history of each relationship matters … It goes yet further, by combining the unique trajectories of all the alters to show how they collectively shape dynamics at the network level.” (Offer et al., 2026: 131).

The approach presents a way of studying dynamic personal networks by analyzing trajectories of alter-ego relationships over time, highlighting their impact on network composition and how personal networks may change in response to life events. A personal network, also called an egocentric network, focuses on the immediate social relationships and reported connections of a single person (the ego/patient), along with their identified connections to various relationships, roles, and intimacy levels (the alters). The sensitivity of personal networks to each alter’s recent history matters. Until recently, personal networks were highly ego-centered, with less attention to alters’ dynamics. In this approach, the trajectory of each alter is emphasized at the network level.

The Alter-Trajectory Approach extends the literature on structural social support and health. According to Offer et al., there are six distinct historical trajectories for alters: continuously active, awakened, dormant, dropped, transitory, and new (Offer et al., 2026). In the study of stressful life events among young people, the most common trajectory was “continuously active” alters in coping with life events (Offer et al., 2026). “Awakened supporters” and “Dropped supporters” were notable as the second- and third-most recurrent trajectory types (Offer et al., 2026). “Transitory supporters” were active at Wave 2 but inactive by Wave 3, whereas “Dormant supporters” were inactive initially but could later transition to active status (Offer et al., 2026). The least common was “New supporters,” which has implications for the addition of novel alters that can add to the support network or replace lost support people, thereby affecting social support (Offer et al., 2026).

When accounting for these diverse trajectories, there are three distinct dynamic network types at the ego level—anchored, shifting, and regenerative—each with unique dynamics and compositional features. According to the Offer et al. study, “Anchored networks” were most common in the young people and stress study, featuring a stable core of active supporters, while “Shifting networks” show significant movement between the core and periphery (Offer et al., 2026). Next, “Regenerative networks” include many new supporters and demonstrate a tendency for the core to shrink over time (Offer et al., 2026). While the Offer et al. young adult cohort under study has its own unique social and physical features, it is hypothesized that the Alter-Trajectory Approach could be used beyond this young adult population, allowing the framework to be tested in other populations.

## 4 Type 2 diabetes- a case for understanding the socio-biological cascade

As an illustration of the application of the Socio-Biological Cascade, social support has wide-ranging implications for conditions such as diabetes. In the United States, it is estimated that 40.1 million people have been diagnosed or undiagnosed with diabetes, with approximately 12% of the population living with the disease (Centers for Disease Control, 2026). It is hypothesized that, to connect the exposome to disease, there must be an intermediary step via the Socio-Biological Cascade; for instance, the social factor of social support can possibly bridge SDOH to Type 2 diabetes health and maintenance.

Notably, “Diabetes is a particularly informative test case for studying exposomes as it has marked social and environmental gradients, showing much higher prevalence of the disorder in poorer, more marginalized populations” (Zia et al., 2026). Considering the exposome’s relevance to the environmental and social contexts of diseases such as diabetes, employing the dynamic interdependencies required by systems thinking aligns it with complexity. The social exposome in Type 2 diabetes has been studied more than that in Type 1 diabetes (Zia et al., 2026). According to Beulens et al., the external exposome of non-genetic, environmental factors can imprint on the internal exposome (Beulens et al., 2022). In terms of implications for systems biology, Type 2 diabetes is a “genotype–environment interaction disease” (Van Ommen et al., 2018).

According to Van Ommen et al., multidisciplinary interventions that are multidimensional in approach are required to reverse diabetes in patients diagnosed with diabetes as a lifestyle-related disorder (Van Ommen et al., 2018). But it should be noted that the social environment in which diabetic patients navigate is also socially complex, which interacts with “lifestyle” in addition to other complex SDOH. Health navigation for patients does not involve only interactions with healthcare providers or is reliant on only personal lifestyle choices. To apply social complexity to such a genotype-environment interaction disease, Van Ommen and colleagues advocate for a 360-degree diagnosis that accounts for the diversity of physical, social, and economic factors affecting the disease, which should be addressed before lifestyle factors (Van Ommen et al., 2018). In this paper, it is argued that the environment associated with lifestyle must incorporate systems thinking to unravel the genotype-environment interaction that leads to lifestyle choices. The complexity of chronic disease management goes beyond lifestyle changes and behavioral choices. Lifestyle is embedded in non-medical social determinants and in the cascade of mediators of social support and social connection.

For instance, in diabetes maintenance, Rad et al. noted that the positive effect of social support for people with diabetes, more specifically, support from spouses and family members, on health-promoting behavior (Rad et al., 2013). Beneficial health-promoting behavior and medication adherence in Type 2 diabetes management have been found to be challenging. Research has shown that having supportive connections, characterized by empathy, acceptance, and concern, may be associated with higher QOL (Park et al., 2023). In a network study taken from the larger Maastricht Study, a smaller network size, a greater proportion of kin, and a lower percentage of friends were associated with macrovascular diabetes complications in both men and women (Brinkhues et al., 2018). Prochnow et al. found that the presence of support in the networks of Black/African American male diabetic patients was more protective of positive diabetes maintenance than network size (Prochnow et al., 2025b). In a study of Mexican immigrants diagnosed with Type 2 diabetes, the greater presence of diabetic alters in one’s network was associated with higher HbA1c (Flórez et al., 2024a). According to Flórez and colleagues, during the COVID-19 lockdown, the presence of diabetic alters in surveyed NYC Mexicans’ social networks also predicted higher HbA1c among the participants (Flórez et al., 2024b).

The complexity of a support person’s own maintenance of diabetes may hamper the quality of social support that can be offered. It may also be possible that positive modeling behavior supports positive diabetes care. Having a greater proportion of diabetes related conversations in surveyed social networks of Black/African American diabetic men was found to be associated with an increased risk of negative diabetic outcomes (Prochnow et al., 2025a). The study noted that this contradictory finding may be associated with increased help-seeking behavior, such as seeking advice from support people in their networks (Prochnow et al., 2025a). According to Crotty et al., diabetic patients with mental illness, with or without a spouse, were more apt to lose support people and present fewer friendship ties due to illness (Crotty et al., 2015). Co-morbidity complicates disease maintenance. The over-reliance on weak ties, such as professional carers, among those without spouses makes these patients’ networks vulnerable to tie instability and more prone to the loss of alters and their social support (Crotty et al., 2015). For diabetic patients with spouses, the presence of a spouse reduced reliance on weak, professionally related ties (Crotty et al., 2015). In addition, social support has been found to have a mediating role in improving diabetes treatment adherence (Erdoğan Yüce and Yıldırım, 2025).

## 5 The concentric model for health-bound networks

**FIGURE 2:**
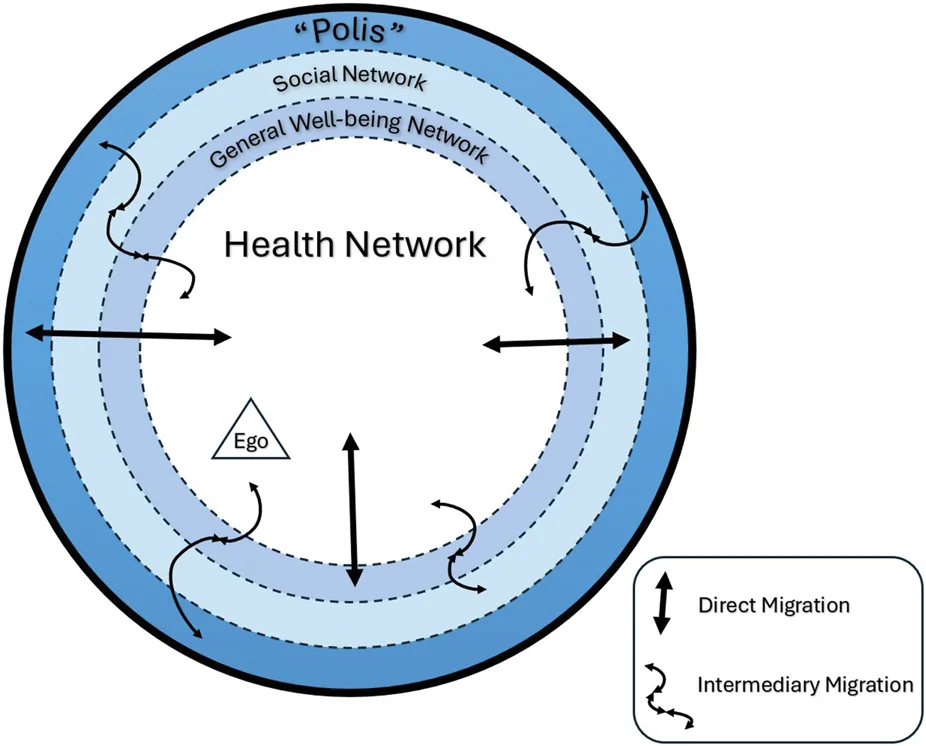
The Concentric Model for Health-Bound Networks’ migration patterns. Reprinted with permission. (Battle-Fisher, 2015).

The Concentric Model for Health-Bound Networks (CMHN) comprises four concentric circles with unique networks that are called spheres (Battle-Fisher, 2015; Dunbar and Spoors, 1995). As illustrated in Figure 2, the spheres in the CMHN can be referred to as the Health Network (1), the General Wellbeing Network (2), the Social Network (3), and the Polis (4). The concentric networks in CMHN center on an ego/patient in an ego network that spans multiple levels, with the inner network (the health network) representing the most central and influential alters (in terms of influential centrality), overlapping alters (called structural folds), and/or substantive-support alters that provide support (Battle-Fisher, 2015). The CMHN boundaries around a sphere are thought to be semi-porous, taking shape and holding their integrity while allowing alters to pass freely across spheres. According to Targarona Rifa et al., such movement can be viewed as the “whole dynamic process of alter (re)location” across bounded personal networks, which in this model compose each concentric sphere (Targarona Rifa et al., 2025). The discussion of temporality in the movement of alters warrants continued study, and the CMHN seeks to investigate it by examining temporal migration patterns across measurement waves. Boundary-making processes are fluid and negotiated with networks. The occurrence of boundary setting in the CMHN is predicted based on the relational connection of social networks and boundary setting and change (Targarona Rifa et al., 2025).

At its core, the Health Network is designed to manage critical medical and wellness concerns, while the General Wellbeing Network focuses on non-medical yet significant life matters. Daily interactions and informal exchanges fall under the Social Network. Finally, the concept of the Polis is introduced as a latent resource that provides no immediate assistance but remains a potential foundation and source for future aid. Together, these categories illustrate a structured approach to identifying where individuals turn for help, depending on the urgency and type of their needs.

Additionally, in Figure 2, the concepts of direct and intermediary migration, represented by the arrows in Figure 2, reflect the movement of support alters across different spheres (Health Network, General wellbeing Network, Social Network, Polis) (Battle-Fisher, 2015). The direct migration is analogous to shifting networks that eventually become stable and anchored across waves (Battle-Fisher, 2015; Offer et al., 2026). In contrast, intermediary networks continually move along a trajectory of spheres and do not reach a final destination over longer durations across waves without intermediate steps, fully skipping spheres to their final destination (Battle-Fisher, 2015; Offer et al., 2026). During migration, it may be possible that any of the supporter categories may apply, though it is hypothesized that the more stable, such as continuously active supporters, may be more dependable over time in their ability to garner social support in the model. It is postulated that a support person may have a role change that necessitates a move. The change across waves proposed by the model does not directly imply a support person’s desire to stay or go. The model, in its current form, indirectly implies multiplex roles that may necessitate a migration of social support over time. The change in role may be due in part to migration, the support person’s capacity or willingness to help, or to a change in the multiplex roles taken on.

Structural folds are support people or alters in a network who take up residence in more than one sphere, thereby showing network embeddedness in cohesive social support systems (De Vaan et al., 2015). According to the CMHN, these structural folds create the overlap between the concentric spheres. According to Vedres and Stark, folds are linked to intercohesion, a term describing the intersection of cohesive groups (Vedres and Stark, 2010). There is a distinction between intercohesion and structural folds: intercohesion describes the overlapping, cohesive network structure, whereas a structural fold is defined by an alter and its overlapping position in intersecting networks (Vedres and Stark, 2010). The structural properties of concentric support networks show that networks can be cohesive and intersecting (Vedres and Stark, 2010).

Based on Dunbar’s circles typology of the support clique, sympathy clique, affinity circle, and active network. Compared with Dunbar’s Circles, CMHN alters may progressively increase in number as spheres enlarge and as relationships form, dissipate, and develop social ties. According to the CMHN, the dissolution of relationships, as well as changes in intimacy and social support, will lead to migration across the spheres (Battle-Fisher, 2015). Alters become more involved, while others assume a more distant supportive role, thereby determining their respective spheres of influence. Therefore, the n’s per sphere are dynamic and subject to migratory change across spheres. It should be noted that each sphere in the CMHN may have its own independent network size. This means there may be diverse forms of social support, which may lead to unique findings on network size over time. If this holds true for the CMHN, smaller, denser inner spheres may be more important than the number of support people in the sphere. It may hold that the quality of supportive ties trumps the size of a health network. According to the CMHN, the most supportive ties populate inner spheres, with the possibility that less involved support people migrate into closer spheres as role intensity changes.

If Dunbar’s Numbers hold true in the CMHN, the Diabetes (Health Network) sphere (as the innermost sphere) may consist of approximately 5 alters, 15 alters to the General wellbeing Sphere, 50 in the Social Network, and 150 expected in the Polis (outermost sphere) (Dunbar and Spoors, 1995; Stiller and Dunbar, 2007; Sutcliffe et al., 2012). Dunbar’s Numbers hypothesize that the number of alters in each sphere (circle) should increase by a multiple of three (5-15-50-150) as the model shows most intimate to less intimate support persons, with a smaller, dense inner circle of alters and the largest population of alters in the least intimate Polis (Sutcliffe et al., 2012). According to the CMHN, the polis is infinite, with possibilities for new alternative connections that can be brought forward; therefore, the n is boundless in theory and not certain. For the Polis boundary to be meaningful, 150 alters may be used in accordance with Dunbar’s Numbers. According to Dunbar’s Numbers, it is assumed that the outermost circle or sphere would have the most alters out of the four in the CMHN model. The application of Dunbar’s Circles is presented as a possible explanation for the CMHN; however, this application is provisional and may change under rigorous validation.

Under the Alter-Trajectory Approach, all distinct trajectories may be at play in the CMHN. It is hypothesized that “continuously active” would be in the innermost sphere (e.g., Diabetes Network) as it includes the most intimate support that directly affects diabetes care. In the CMHN, “awakened supporters” may be evidenced by increased and emergent diabetes needs, calling for alters to take on new roles while remaining in their current sphere. When support people migrate closer to or farther from the center of the concentric circles after a period of dormancy, they may still be reported by the ego as a support person even when they are not currently involved. Involvement in support may be linked to the actual, probable, and inactive to active support of an alter. It is hypothesized that more casual supporters would be the source of “new supporters”. The polis, because of its current lack of social relationships, intimate or casual to the ego, may be the least likely to activate and become a supporter. An exception may be professional support personnel, such as medical professionals, who may enter the network with no historical tie to the patient at different times.

**FIGURE 3:**
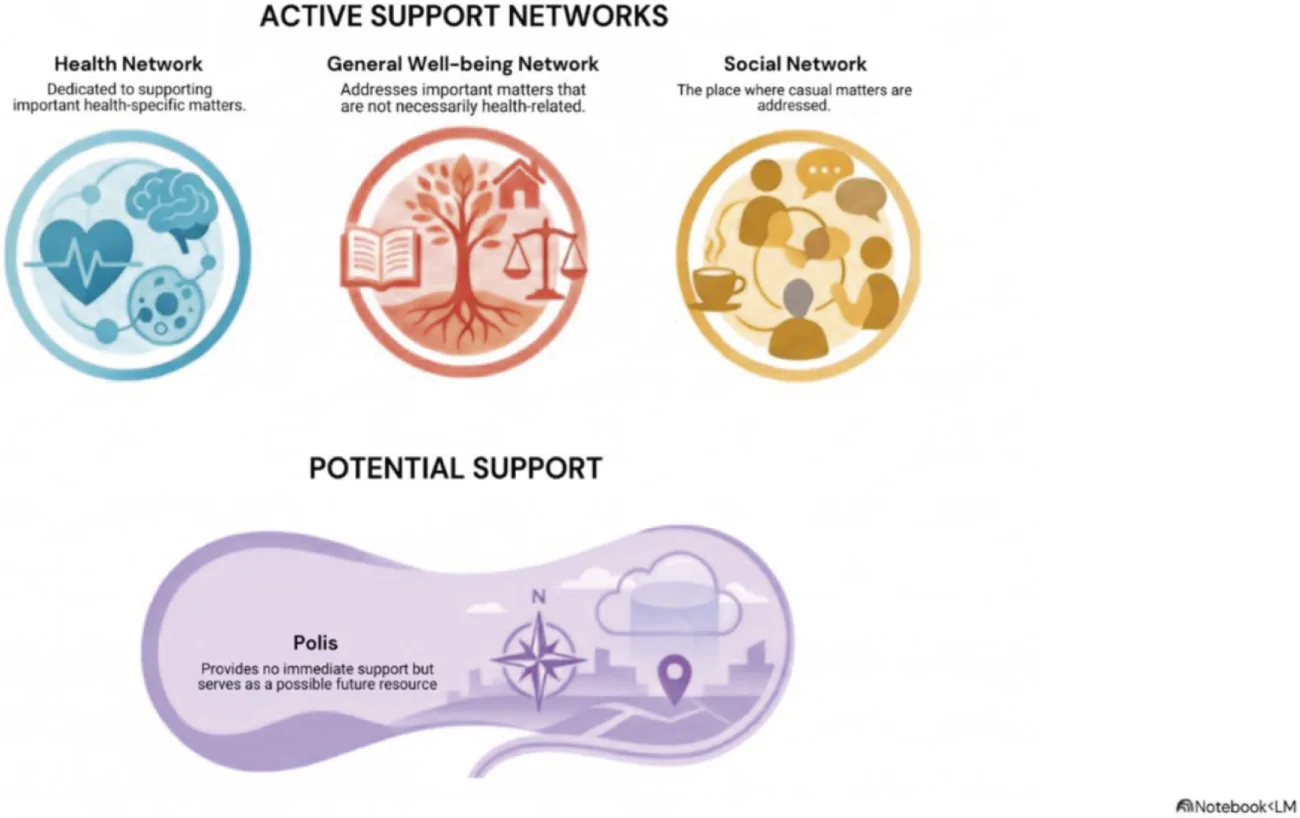
The Concentric Model for Health-Bound Network Spheres with “Health Matters” Framing. Figure created using AI (Gemini Notebook). (Battle-Fisher, 2015; Perry and Pescosolido, 2015).

Perry and Pescosolido noted that, in accordance with the Functional Specificity Hypothesis, people selectively activate social connections or ties for productive purposes or for positive, fruitful exchange of goods (Perry and Pescosolido, 2010). The “Important matters” and “health matters” navigation options in social networks are linked to the distribution of needs and support sources, as care-intensive illness requires more than one person to meet the patient’s needs (Perry and Pescosolido, 2010). Perry and Pescosolido found that characteristics of the “health matters” networks significantly affected health outcomes (Perry and Pescosolido, 2010). Cassel famously linked network-based social support to illness risk, and Cobb proposed that strong social ties are protective against stressful events (Cassel, 1976; Cobb, 1976). As health-bound social networks evolve, patients’ needs respond to social forces throughout the ego’s illness career, with the illness career defined as “a variety of fluid pathways that individuals and their social networks follow in response to illness” (Perry and Pescosolido, 2015: 2). Applying the Network Episode Model (NEM), health-bound networks can be described in terms of structure, function, and content (Perry and Pescosolido, 2015). These attributes of social network organization are influenced by dynamic, personalized experiences with illness (Perry and Pescosolido, 2015). Failing to examine illness careers and network ties in relation to help-seeking behavior leaves a gap in understanding how social networks are activated and maintained. In an adaptation of the earlier conceptualization of the model with Perry and Pescosolido’s framing of “important matters” and “health matters” added, the CMHN’s concentric, overlapping spheres can be reimagined as follows in Figure 3:

Falvey and colleagues’ “circles of support” model includes four circles: the innermost circle of intimacy; the circle of friendship; the circle of participation; and the circle of exchange (All My Life’s a Circle: Using the Tools: Circles, MAPS & PATHS, 2003). Unlike the CMHN, the “circles of support” framework places the circle of exchange at the outermost level and includes both paid support, such as health professionals, and general support, such as housecleaning (All My Life’s a Circle: Using the Tools: Circles, MAPS & PATHS, 2003). This conceptualization is a professional service exchange rather than formal or informal social support for the largest, outermost circle. In the proposed CMHN model, the medical professional would be intimately connected to support people in the innermost sphere, with frequent contact regarding illness and kin or intimate non-kin involved in diabetes (health)-related care and support. Therefore, the Diabetes (Health) Sphere most likely includes medical professionals in the innermost sphere, which is contrary to the “circles of support” framework (All My Life’s a Circle: Using the Tools: Circles, MAPS & PATHS, 2003; Battle-Fisher, 2015).

In cultural and organizational contexts, context permeability refers to simultaneous actor interaction across different social networks, with interaction among networks (Nunes and Antunes, 2015). Nunes and Antunes present another distinct model called “context switching”, where the temporal element of moving across different co-existing networks occurs (Nunes and Antunes, 2015). This context switching can inform direct and intermediary migration within the CMHN, as actors may continue to move between and across coexisting networks, spending varying amounts of time in each based on intimacy, changes in support giving, and multiplex roles (Nunes and Antunes, 2015). As social support roles and interactions with the patient change for the support person, that person’s placement in a new sphere also changes. This dynamic movement highlights the idea of boundaries and their permeability.

## 6 Testing the concentric model for health-bound networks and the socio-biological cascade

The CMHN is rooted in the dynamic movement of alters across the concentric spheres of support and influence on the ego/patient in question. The Socio-Biological Cascade attempts to elucidate the interdependence of SDOH, social support, and physiological disease and its implications for health outcomes. Ultimately, this Socio-Biological Cascade from society-to-biology may in part determine an individual’s long-term physical wellbeing.

To test the Socio-Biological Cascade, connections of SDOH to social support and connections of social support to relevant biological indicators, such as cortisol, inflammatory markers, HbA1c, or allostatic-load measures, are required. Existing, psychometrically validated social support survey instruments such as the All of Us Research Program Social Determinants of Health survey, whose measures include emotional and instrumental social support, perceived stress, as well as a variety of other SDOH can be used (Tesfaye et al., 2024). In addition to these surveys, an SNA survey (including a name generator) is required to map the nature of ego-alter network structure and dynamics for SNA analysis and visualization. To test social support for biological processes, methods such as prospective and longitudinal surveys, as well as qualitative methods, apply. Psychophysiological and other physiological methods measure physiological responses to external stimuli. Wearable health devices have been used to track body changes, which have been used to understand and track stress levels and their implications for stress management (González Ramírez et al., 2023). Advances in genomics and the study of gene expression across the life course also hold promise in exploring the biosocial effects of the exposome (McDade and Harris, 2022).

While not currently included in the external exposome debate, the CMHN highlights that the social environment encompasses socially complex factors, such as SDOH, that can influence the lifestyle environment of the external exposome. To test the hypotheses presented by the CMHN, SNA methods and measures can be utilized for this purpose. This includes measuring and mapping relevant social network variables, such as network size, density, tie strength, and alter migration via structural folds.

In the CMHN, there is the opportunity to be an alter who spans social network spheres, vested with a folding position that entails intimate knowledge of multiple groups (Vedres and Stark, 2010). In accordance with structural folds, there are alters that span across groups/spheres. According to Vedres and Stark, there are four parts to the Structural Fold Methodology:

1. Identification of cohesive groups, such as those with overlapping membership.
2. Visualize the intercohesion of members (via membership in more than one group) using network maps.
3. Analyze intercohesion via evaluation of sharing, access, or a combination of goods and resources based on the position of shared membership.
4. “Temporal Analysis” (also called Generative Disruption) via tracking “separation and reunification” of groups longitudinally to assess evolution of systems. (Vedres and Stark, 2010; 2012).

This Structural Fold Methodology enables the CMHN to discover membership overlaps, which is central to the model. In the model, it is hypothesized that there are four levels of spheres, each with its own distinctive nature of social support. The folds that connect the spheres experience intercohesion, which may be sources of both generative disruption and innovation (Vedres and Stark, 2010).

The presence of direct and intermediary migration aligns with generative disruption and its analysis, as it illustrates changes in the structural composition of each sphere over time. The Alter-Trajectory Approach accounts for both longitudinal waves to examine waves over time and dynamic change in alters in networks (Offer et al., 2026). The approach accounts for this using a longitudinal social network survey to map for “new”, “lost” and “reappearing” alters across waves (Offer et al., 2026). An alter-trajectory measure is required, capturing “past, current and subsequent” relationships with ego across waves (Offer et al., 2026). In addition, in order to label spheres using CMHN using the Alter-Trajectory Approach, questions could be asked with the SNA survey to label each alter listed into a sphere at each wave. The mapping of new, lost, and reappearing alters can be used in tandem. The Alter-Trajectory Approach study uses Multilevel Latent Growth Models (MLGM) to measure, and label alter groups by trajectory based on repeated measures.

The implications for systemic biological and social contexts in chronic disease are great. The prevalence of this chronic disease has implications for the overall health and wellbeing of people who navigate its complex care requirements. Possible health outcomes include disease control, resilience, quality of life (QOL), and treatment adherence. Methods such as prospective and longitudinal surveys, cohort studies, clinical trials, and qualitative methods can be used to explore society-to-biology research questions.

## 7 Limitations of the model

Conceptual models, such as the CMHN, have limitations. CMHN is conceptual and has not been validated with real-world data and scenarios. Measurement is key to decoding and validating relationships between biological and social phenomena. The model requires empirical validation, and potential biases may exist. It is acknowledged that conceptual models may oversimplify real systems and that care must be taken when extrapolating their application to social situations. In addition, the use of a socio-biological framework such as the Socio-Biological Cascade does not imply causation across SDOH, social networks, and biological processes. Social support mechanisms can act as mediators or moderators of biological processes, supporting the development of the model. Frameworks offer generalizations of how complex processes may work. The model rests on ego-reporting, also known as egocentric bias, which may be affected by recall bias, perceptions of support bias, and network centrality bias, in which alters misrepresent their influence within the network or the accuracy of reported connections (Johnson and Orbach, 2002). It is further acknowledged that ego-reporting may not be a true proxy for actual network structure and behavior. In addition, there is early work on longitudinal measures of social networks. Limitations in longitudinal measurement may include alters’ gains and losses not being accurately captured by ego-reporting. Another limitation is that the alters in the model, as conceptualized, do not report their statuses in the CMHN. Lastly, the assertions of Dunbar’s Circles are extrapolated to a different scenario than originally measured in Dunbar’s work. Also, the uses of Dunbar’s Circles and the Alter-Trajectory Approach are provisional in their application to the CMHN. Network structure may be influenced by the meaning of support, which varies across cultures, communities, and stages of illness. As presented by the social-elastic tie, social relationships can generate strain, conflict, and physiological burden, as well as perceived and actual social support. Lastly, longitudinal data are required to identify patterns and the durability of social support configurations and migratory changes.

## 8 Discussion

Social support has been shown to have significant implications for the care of patients with chronic disease and for their health outcomes. Palmer and colleagues posit that embodiment, which illustrates “how adverse exposures affect the body and how they lead to illness and disease … which gets under the skin,” is understudied and should be undertaken in research (Palmer et al., 2019: S70). In this quest to make sense of the embodiment connection between the body and health outcomes, there is room for evidence directly linking biology to societal exposure (Palmer et al., 2019). There is potential to link SDOH to social support, which has been shown to elicit physiological responses in biological systems. The CMHN centers embodiment as a central element that reveals how health effects for patients may be connected to social connections and the social support they provide or do not provide. In terms of what contributes to systemic forces that lead to unequal effects within different complex systems and social networks, “The system’s mechanism is not to blame, as it acts mechanically according to its nature and purpose … the social implications of the system’s behavior are crucial” (Battle-Fisher, 2026: 3). The objectives of the theoretical systems modeling hypothesis exercise were to:

1. Explore how the complexity of maintaining and navigating social support for care-intensive health concerns affects an individual ego patient using social network analysis and systems theory.
2. Explore whether complex network-based models, such as the one presented, can accurately describe the migration dynamics of support people and the changing nature of roles in support across various levels of social support.
3. Explore the model’s ability to uncover the effects of dynamic social support during complex illness.
4. Explore the application of trajectories of alters using the “Alter-Trajectory Approach” as a guide to understanding ego network dynamics.
5. Highlight the connections between biology, SDOH, and social support in the Socio-Biological Cascade.

Social support in the external exposome consists of networks of people who help patients with diverse health needs, and those networks in the environment influence health. Non-medical SDOH affects both the state of illness and the nature and need for social support. The CMHN applies network science, combined with public health and sociological frameworks, to explore support for care-intensive illnesses and their physiological effects. The model calls for a complexity-driven reimagining of social support for complex illnesses, accounting for interconnections among and with social determinants that may lead to poorer outcomes and diminished quality of life. In the CMHN, navigating the complex nature of social bonds may place greater demands on the social brain, which tests a patient’s neurology in handling illness-related stress. As the social brain is evolutionary, so are the dynamics of social support to health. Support, as well as disease states, is not static, which raises the interesting question of how changes in social safety and the social brain play out alongside complex social changes.

It is proposed that there are several future directions in employing CMHN to understand dynamic social support and physiological health:

1. How do ego networks develop, and how resilient are they over time, considering dynamic social support-based migration patterns?
2. Do Dunbar’s Circles’ progression and reduction patterns in network size and levels of intimacy hold in the CMHN?
3. How can the Socio-Biological Cascade inform our understanding of complex illness maintenance and navigation?
4. How can CMHN and the Socio-Biological Cascade be leveraged for genotype-environment interaction diseases, such as Type 2 diabetes?
5. What can be learned about the social brain and social safety using the CMHN?

There is an opportunity to explore health using systems biology, social support, and patients’ health needs, as mediated through overlapping social networks. Utilizing CMHN as a framework using social network analysis offers a novel way to understand social support as an element of a socio-biological cascade that supports or impedes health outcomes. There is potential in exploring systems biology and the complexity of social support networks for patients with support needs. The paper outlines the state of the social support literature, the public health issues facing, for example, Type 2 diabetic patients, the application of network science to social support, and a refinement of the CMHN model. This paper presents an approach that uses CMHN as a framework for understanding a path to mitigating the global burden of chronic illnesses and highlights its biological complications. As evidenced by the Type 2 diabetes discussion, understanding this socio-biological complexity has major implications for clinical care and public health. Social forces affect structural levels of health, as reflected in the incidence and prevalence of disease. The model provides a basis for operationalizing the complexity of social support using network science and systems biology, a novel approach in health and medicine.

## Data Availability

The original contributions presented in the study are included in the article/supplementary material, further inquiries can be directed to the corresponding author.
